# Mechanical versus manual chest compressions in the treatment of in-hospital cardiac arrest patients in a non-shockable rhythm: A multi-centre feasibility randomised controlled trial (COMPRESS-RCT)

**DOI:** 10.1016/j.resuscitation.2020.09.033

**Published:** 2021-01

**Authors:** Keith Couper, Tom Quinn, Katie Booth, Ranjit Lall, Anne Devrell, Barry Orriss, Scott Regan, Joyce Yeung, Gavin D. Perkins

**Affiliations:** a Warwick Clinical Trials Unit, Warwick Medical School, University of Warwick, Coventry, UK; b University Hospitals Birmingham NHS Foundation Trust, Birmingham, UK; c Emergency, Cardiovascular and Critical Care Research Group, Faculty of Health, Social Care and Education, Kingston University, London and St George’s, University of London, London, UK; d Patient Research Ambassador

**Keywords:** Cardiac arrest, Advanced life support, Cardiopulmonary resuscitation, Mechanical chest compressions, Randomised controlled trial, Feasibility trial

## Abstract

**Background:**

Mechanical chest compression devices deliver high-quality chest compressions. Early data suggests that mechanical devices may be superior to manual chest compressions in adults following an in-hospital cardiac arrest patients. To determine the feasibility of undertaking an effectiveness trial in this population, we undertook a feasibility randomised controlled trial.

**Methods:**

We undertook a multi-centre parallel group feasibility randomised controlled trial (COMPRESS-RCT). Adult in-hospital cardiac arrest patients that were in a non-shockable rhythm were randomised in a 3:1 ratio to receive mechanical CPR (Jolfe AB/Stryker, Lund, Sweden) or ongoing manual CPR. Recruitment was led by the clinical cardiac arrest team.

The primary study outcome was the proportion of eligible participants randomised in the study during site operational recruitment hours. Patients were enrolled under a model of deferred consent. We report data using descriptive statistics, point estimates and 95% confidence intervals.

**Results:**

Over a two-year period, we recruited 127 patients across five UK hospitals. We recruited 55.2% (95% CI 48.5%–61.8%) of eligible study participants in site operational recruitment hours. Most participants were male (n = 76, 59.8%) with a mean age of 72 (95% CI: 69.9–74.9) years. Median arrest duration was 18 (IQR 13−29) minutes. In patients randomised to mech-CPR, median time from CPR start to device deployment was 11 (IQR 7−15) minutes. ROSC was achieved in 27.6% (n = 35) participants and 4.7% (n = 6) were alive at 30-days.

**Conclusion:**

COMPRESS-RCT identified important factors that preclude progression to an effectiveness trial of mechanical CPR in the hospital setting in the UK. Findings will inform the design of future in-hospital intra-arrest intervention trials.

ISRCTN38139840, date of registration 9th January 2017.

## Background

In the UK, there are approximately 35,000 in-hospitals cardiac arrests per year with an overall hospital survival of 18.4%.^1^ High-quality cardiopulmonary resuscitation (CPR), defibrillation, and reversal of the underlying cause are the mainstay of cardiac arrest treatment.^2^ However, delivery of high-quality CPR in clinical practice is often challenging.^3^, ^4^, ^5^

Mechanical chest compression devices (mech-CPR) deliver high-quality CPR.^6^ In out-of-hospital cardiac arrest, large randomised controlled trials show that mech-CPR is not superior to manual CPR (man-CPR).^7^, ^8^, ^9^ As such, current guidelines recommend against the routine use of mech-CPR.^10^ In contrast, research on mech-CPR use in the hospital setting has been limited. Small randomised controlled trials and observational studies have produced very low-certainty evidence that mech-CPR use in the hospital setting is associated with improved clinical outcomes.^11^

Based on evidence of mech-CPR use at in-hospital cardiac arrest and uncertainty regarding its effectiveness, we identified a need for a clinical trial to evaluate the effect of the routine use of mech-CPR, compared with man-CPR in adults that sustain an in-hospital cardiac arrest.^12^, ^13^ In contrast to out-of-hospital cardiac arrest, there have been relatively few trial of intra-arrest interventions for in-hospital cardiac arrests.^14^ As such, we decided to first test the deliverability of a mech-CPR trial in a feasibility trial.

## Methods/design

We conducted a multi-centre parallel group randomised controlled feasibility trial across five UK hospitals. We randomised in a 3:1 ratio to either mech-CPR or man-CPR. The trial objective was to assess how feasible it would be to deliver an effectiveness mech-CPR trial in the in-hospital cardiac arrest population.

The trial was approved by the West Midlands – Coventry and Warwickshire Research Ethics Committee (16/WM/0299). The Health Research Authority Confidentiality Advisory Group approved the processing and transfer of data without consent, under The Health Service (Control of Patient Information) Regulations 2002 (16/CAG/0088). We prospectively registered the trial with the ISRCTN Trial Registry (ISRCTN08233942). We published the protocol in an open-access journal.^15^ A National Institute for Health Research Post-Doctoral Research Fellowship (PDF-2015-08-109) funded the trial.

We conducted in accordance with Medical Research Council (MRC) Good Clinical Practice guidelines, national legislation and University of Warwick Clinical Trials Unit Standard Operating Procedures. The University of Warwick sponsored the trial.

During the trial, we amended the primary outcome from proportion of eligible patients randomised to proportion of eligible patients randomised during operational recruitment hours. This change was made on 21st March 2018 (midway through trial recruitment) due to the challenges experienced by some sites in recruiting 24/7. Our ISCTRN registration records this change.

### Eligibility criteria

Adults (age ≥18) that sustained an in-hospital cardiac arrest were eligible for inclusion if the cardiac arrest was attended by an emergency team trained in the use of mech-CPR and the patient was in a non-shockable rhythm at the point of the study eligibility assessment. Key exclusion criteria included known pregnancy, prisoners, known previous study participation, and cases where mech-CPR was contraindicated (for example, patient size) or required as part of routine clinical care (for example, cardiac arrest during coronary angiography).

We defined in-hospital cardiac arrest to exclude events in the emergency department. We excluded patients in a shockable rhythm due to a finding of harm associated with mech-CPR use in this patient group in the PARAMEDIC trial.^7^ This may be attributed to delays in defibrillation in the mech-CPR group, although it has not been replicated in other trials.^16^, ^17^

For a team to be considered trained in the use mech-CPR, at least two clinicians were required to be competent in device use. This safeguard was implemented as previous research has highlighted that mechanical chest compression device deployment can be associated with prolonged chest compression pauses.^18^, ^19^ We took the view that ensuring at least two people present were competent in the deployment process would mitigate this risk. Our approach was informed by our preparatory simulation work.^20^

### Study interventions

Following confirmation of cardiac arrest, all patients received man-CPR. Following randomisation, the cardiac arrest team deployed the mechanical chest compression device (mech-CPR- intervention group) or continued to deliver manual CPR (man-CPR- control group). All other treatments were delivered in accordance with Resuscitation Council (UK) guidelines.^21^

A LUCAS-2 or LUCAS-3 mech-CPR device (Jolfe AB/Stryker, Lund, Sweden) was deployed as soon as possible following randomisation in participants randomised to mech-CPR. Teams were trained using a pit-stop approach to minimise pauses in chest compression delivery during device deployment through use of a two-stage deployment process.^20^ The target maximum chest compression pause during each phase of deployment was ten seconds.

In patients randomised to the man-CPR arm, participants continued to receive manual chest compressions. If available, teams were permitted to use a real-time audiovisual feedback device to guide man-CPR delivery.

### Outcome measures

The primary outcome was the proportion of eligible patients randomised during site operational recruitment hours.

Secondary outcomes included a range of measures, grouped as study feasibility outcomes, patient outcomes, process outcomes, and safety outcomes (Table S1 in Supplementary material). Our patient follow-up included an assessment of survival, neurological outcome and quality of life at six-months. Patient outcomes were selected to comply with the Core Outcome Set for Cardiac Arrest (COSCA) statement.^22^

### Recruitment and randomisation

We designed the trial to facilitate recruitment 24-h a day by the hospital cardiac arrest team.

On arrival of the mechanical chest compression device at the cardiac arrest event, a trained clinician assessed patient trial eligibility. Eligible patients proceeded to randomisation. We used a sequentially numbered sealed opaque tamper-proof envelope randomisation system. A single envelope was stored with each trial device. We randomised eligible patients on an individual basis in a 3:1 ratio in favour of the use of the mech-CPR.

We used an unequal randomisation ratio to increase clinician’s potential exposure to mech-CPR use as this would better correlate with exposure if devices were implemented in practice. Our expectation was that this would help to optimise the deployment process and safeguard participants against the potential harm associated with prolonged chest compression pauses during mechanical chest compression device deployment.

At the point that an envelope was opened, we categorised the participant as being randomised for the intention-to-treat analysis. One randomisation envelope was stored with each mechanical device. Following envelope use, the next sequentially numbered envelope was allocated to that device. The study statistician generated the randomisation sequence, using the centre as strata and random block sizes to ensure that a 3:1 allocation was maintained for each strata. A staff member at the trial co-ordinating centre, who was independent of the study team, packed the envelopes.

### Blinding

We ensured allocation concealment through the use of an opaque envelope system. We were unable to blind the clinical team as they were required to deliver the clinical intervention. We did not specifically seek to blind all site research teams as the randomisation details were recorded in the patient’s medical record, but requested that a blinded researcher support the participant to complete discharge questionnaires. We also did not blind staff at the trial co-ordinating centre as knowledge of allocated intervention was required for monitoring of compliance. Participants were initially blinded as they would be unconscious due to cardiac arrest. We measured blinding success through study questionnaires in which survivors were asked if they were aware of their allocated treatment intervention.

### Consent

Patients were enrolled in the trial under a deferred consent model, as approved by a Research Ethics Committee, in accordance with English law. We approached participants, or a surrogate decision maker, at the earliest reasonable opportunity following the cardiac arrest event to seek consent for ongoing data collection.

### Sample size and statistical analysis

We planned to recruit for a period of two-years or until we reached 330 participants, whichever came first. Feasibility trials typically recruit 25–50 patients per study arm.^23^ Our planned target of 330 participants was to ensure sufficient precision in our estimate for the primary outcome and to use the Cocks and Torgerson approach to determine the statistical appropriateness of progression to an effectiveness trial.^24^ For our trial, we estimated a sample size for an effectiveness trial of 3554 patients, based on detecting a 3.5% absolute improvement in 30-day survival at a power of 90% and a significance level of 0.05. As such, we determined that 330 patients were required for this feasibility trial (9% of 3554), after accounting for loss to follow-up.^24^

For our statistical analysis, we describe categorical data as frequency and percentage and continuous data as mean and standard deviation or median and interquartile range, depending on normality of the data distribution. In our statistical plan, we described plans to compare group outcomes, as we would for an effectiveness trial, by describing risk ratio and 95% confidence interval or mean difference and 95% confidence interval, as appropriate. In addition, for the outcome of 30-day survival, we planned to compare groups using an 80% one-sided confidence interval, as described above. Analyses are undertaken on an intention-to-treat basis.

## Results

Over a two-year period (February 2017 to February 2019), COMPRESS-RCT ran at five UK hospitals, of which three hospitals recruited patients on a 24/7 basis. Sites screened a total of 936 cardiac arrests, of which 662 occurred during site operational recruitment hours (figure one). After excluding 432 events for patient reasons, we randomised 127 (99 mech-CPR; 28 man-CPR) out of 230 potentially eligible patients in cardiac arrest.

The proportion of patients randomised during operational recruitment hours was 55.2% (95% confidence interval 48.5%–61.8%). In total, 38.6% participants were randomised outside normal office hours and 74.8% participants had analysable CPR quality data (Table 1; Table S2 in Supplementary material). Some feasibility outcomes, such as blinding success, are challenging to interpret due to the low number of participants that reached that part of the trial.

**Table 1:** Study feasibility outcomes.

	Outcome
Proportion of eligible patients randomised during site operational recruitment hours	55.2% (95% CI 48.5–61.8)
	n = 127 of 230
Proportion of patients randomised outside of working hours	38.6% (95% CI 30.1–47.6)
	n = 49 of 127
Proportion of patients/consultees agreeing to ongoing study participation	77.8% (95% CI 40.0–97.2)
	n = 7 of 9
Percentage of patients with analysable chest compression quality data	74.8% (95% CI 66.3–82.1)
	n = 95 of 127

The mean age of participants was 72 (95% CI: 69.9–74.9) years and 59.8% (n = 76) were male (Table 2, Table 3). Most were medical in-patients (n = 90, 70.9%) with an initial rhythm of pulseless electrical activity (n = 77, 60.6%). Using the GO-FAR score, most participants (n = 80, 63.0%) were estimated to have 3–15% (average) chance of survival with good neurological outcome based on pre-arrest factors.^25^

**Table 2:** Participant characteristics.

		Mech-CPR (n = 99)	Man-CPR (n = 28)	All cases (n = 127)
Age (years) – mean (95% CI)		72 (69.5–75.1)	73 (67.1–78.4)	72 (69.9–74.9)
Sex-male – n(%)		60 (60.6)	16 (57.1)	76 (59.8)
Weight (kg) – mean (95% CI)		71.5 (67.7–75.3)	77.6 (69.6–85.7)	73.0 (69.5–76.4)
Height (cm) – mean (95% CI)		167.5 (164.8–170.3)	167.3 (162.7–172.0)	167.5 (165.2–169.8)
Baseline CPC – median (IQR)		1 (1−2)	1 (1−1)	1 (1−1)
Patient category – n(%)				
	Trauma	9 (9.1%)	2 (7.1%)	11 (8.7%)
	Medical	72 (72.7%)	18 (64.3%)	90 (70.9%)
	Elective/scheduled surgery	8 (8.1%)	2 (7.1%)	10 (7.9%)
	Emergency/urgent surgery	9 (9.1%)	6 (21.4%)	15 (11.8%)
	Outpatient	1 (1.0%)	0 (0.0%)	1 (0.8%)
Go-far score-likelihood of survival with good neurological outcome – n(%)				
	Very low	4 (4.0%)	0 (0.0%)	4 (3.2%)
	Low	16 (16.2%)	5 (17.9%)	21 (16.5%)
	Average	64 (64.7%)	16 (57.1%)	80 (63.0%)
	Above average	15 (15.2%)	7 (25.0%)	22 (17.3%)

Missingness – weight 12 cases (11 mechanical, 1 manual); height 29 cases (24 mechanical, 5 manual).

**Table 3:** Cardiac arrest characteristics.

		Mech-CPR (n = 99)	Man-CPR (n = 28)	All cases (n = 127)
Initial rhythm – n(%)				
	PEA	59 (59.6%)	18 (64.3%)	77 (60.6%)
	Asystole	36 (36.4%)	10 (35.7%)	46 (36.2%)
	VF/VT	4 (4.0%)	0 (0%)	4 (3.2%)
Rhythm at time of randomization – n(%)				
	PEA	60 (60.6%)	18 (64.3%)	78 (61.4%)
	Asystole	37 (37.4%)	10 (35.7%)	47 (37.0%)
	VF/VT	2 (2.0%)	0 (0%)	2 (1.6%)
Arrest monitored – n(%)		42 (42.4%)	14 (50.0%)	56 (44.1%)
Arrest witnessed – n(%)		62 (62.6%)	17 (60.7%)	79 (62.2%)
Cardiac arrest location n(%)				
	Ward/Emergency Admissions Unit	84 (84.8%)	24 (85.7%)	108 (85.0%)
	Coronary Care Unit	4 (4.0%)	1 (3.6%)	5 (3.9%)
	Critical Care Unit	3 (3.0%)	2 (7.1%)	5 (3.9%)
	Imaging Department	3 (3.0%)	1 (3.6%)	4 (3.1%)
	Specialist Treatment Area	2 (2.0%)	0 (0%)	2 (1.6%)
	Other	3 (3.0%)	0 (0%)	3 (2.4%)
Surface where CPR performed – n(%)				
	Foam mattress	59 (59.6%)	18 (64.3%)	77 (60.6%)
	Air mattress	23 (23.2%)	5 (17.9%)	28 (22.1%)
	Floor	6 (6.1%)	4 (14.3%)	10 (7.9%)
	Other	6	1 (3.6%)	7
	Unknown	5 (5.1%)	0 (0%)	5 (3.9%)
Mechanical device used – n(%)		71 (71.7%)	0 (0%)	71 (55.1%)
Adrenaline				
	Administered – n(%)	99 (100%)	26 (92.9%)	125 (98.4%)
	Dosage (mg) – median (IQR)	3 (2−5)	3 (2−4)	3 (2−4)
Type of advanced airway – n(%)				
	Tracheal tube	33 (33.3%)	11 (39.3%)	44 (34.7%)
	Supraglottic airway	50 (50.5%)	15 (53.6%)	65 (51.2%)
	Not used	16 (16.2%)	2 (7.1%)	18 (14.2%)
Arrest timings – median (IQR)				
	Arrest duration (CPR start to CPR stop)	19 (13−30)	18 (10−25)	18 (13−29)
	CPR start to mechanical device arrival	6 (3−9)	4 (2−6)	5 (3−8)
	CPR start to randomisation	7 (4−11)	5 (3−7)	6 (4−10)
	CPR start to mechanical compressions	11 (7−15)	–	11 (7−15)

PEA – pulseless electrical activity; VF/VT – ventricular fibrillation/ventricular tachycardia.

Missingness – witnessed 2 cases (2 mechanical); adrenaline dosage 3 cases (3 mechanical); arrest duration 4 cases (4 mechanical); time to device arrival 5 cases (5 mechanical); time to randomisation 4 cases (4 mechanical); time to first mechanical compression 3 cases (3 mechanical).

**Fig. 1:**
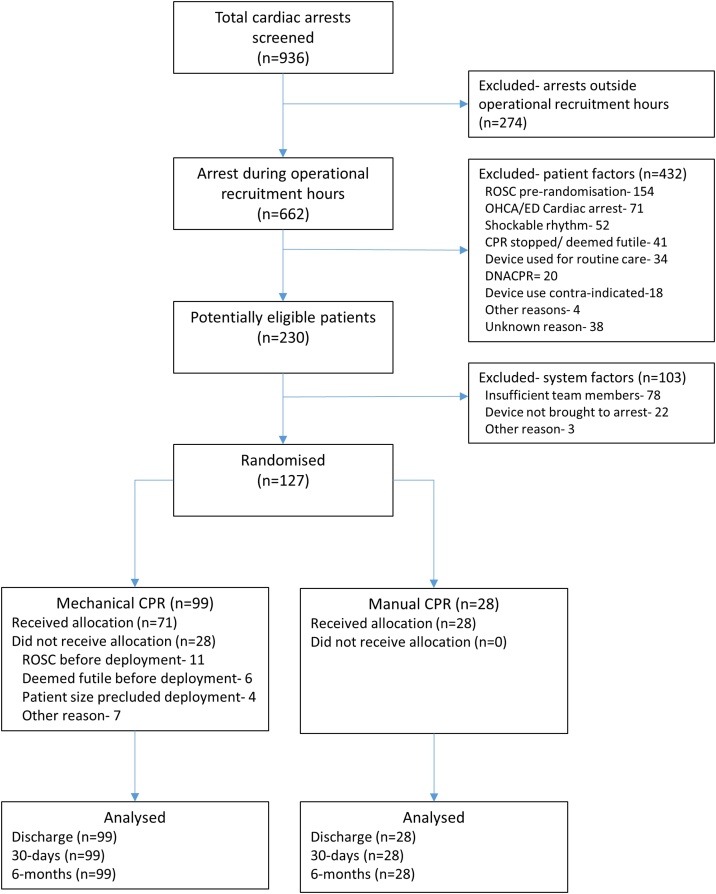
Trial CONSORT flow chart.

Median arrest duration was 18 (IQR 13−29) minutes, with median time from CPR start to randomisation of 6 min (IQR 4−10). In patients randomised to mech-CPR, median time from CPR start to device deployment was 11 (IQR 7−15) minutes. In the 99 patients randomised to mech-CPR, 71.7% (n = 71) received mechanical chest compressions. The main reason for not using mech-CPR was return of spontaneous circulation prior to deployment (Fig. 1).

Data on mechanical device deployment were available for 62.9% (n = 44) patients that received mech-CPR (Table 4). Mean time to deploy the device back plate and upper unit of the device was 7.4 (95% CI 6.0–8.9) and 9.8 (95% CI 7.9–11.8) seconds respectively. CPR quality in both groups adhered, on average, to current guidelines (Table 4; Table S3 in Supplementary material).^26^

**Table 4:** CPR quality and device deployment metrics.

		Mech-CPR (n = 99)	Man-CPR (n = 28)	All cases (n = 127)
CPR quality – mean (95% CI)				
	Compression depth (mm)^a^	59.2 (56.0–62.3)	59.1 (49.5–68.8)	59.2 (56.0–62.3)
	Compression rate (/min)	109.0 (107.4–110.7)	115.6 (110.5–120.6)	110.4 (108.7–112.1)
	Flow-fraction	0.86 (0.9–0.9)	0.89 (0.9–0.9)	0.87 (0.9–0.9)
Device deployment – mean (95% CI)				
	Pause for backplate (seconds)	7.43 (6.0–8.9)	–	–
	Pause for upper part of device (seconds)	9.83 (7.9–11.8)	–	–
	Flow fraction in minute preceding first mechanical compression	0.68 (0.6–0.7)	–	–

Missingness – CC depth 61 cases (48 mechanical, 13 manual); CC rate 32 cases (24 mechanical; 8 manual); flow-fraction 32 cases (24 mechanical; 8 manual); device deployment 27 cases.

a CC depth in mech-CPR arm describes CC depth prior to device deployment. CC depth in man-CPR describes depth over entire event.

ROSC was achieved in 27.6% (n = 35) participants (Table 5). Survival to discharge, 30-days and six-months was observed in 3.9% (n = 5), 4.7% (n = 6), and 3.1% (n = 4) participants respectively. All five patients that survived to hospital discharge had a good neurological outcome (n = 5, 3.9%). Length of stay and quality of life outcomes are reported in Table S4 in the Supplementary material.

**Table 5:** Participant outcomes.

			Mech-CPR (n = 99)	Man-CPR (n = 28)	All cases (n = 127)
ROSC≥ 20 min – n(%)			28 (28.3%)	7 (25.0%)	35 (27.6%)
Survival – n(%)					
	Hospital discharge – n(%)		4 (4.0%)	1 (3.6%)	5 (3.9%)
	30 days – n(%)		5 (5.1%)	1 (3.6%)	6 (4.7%)
	6-month – n(%)s		3 (3.0%)	1 (3.6%)	4 (3.1%)
Survival with good neurological outcome (CPC) n(%)					
	Discharge		4 (4.0%)	1 (3.6%)	5 (3.9%)
Survival with good neurological outcome (mRS) n(%)					
	Discharge		4 (4.0%)	1 (3.6%)	5 (3.9%)
	6-months		1 (1.0%)	0 (0.0%)	1 (0.8%)

ROSC – return of spontaneous circulation; CPC – cerebral performance category; mRS – modified Rankin score.

Missingness – mRS at 6-months 2 cases (1 mechanical; 1 manual).

Four device adverse events were reported, of which none were categorised as serious. In two cases, the device did not start and both events were attributed to human error. In one case, the device did not restart after a rhythm assessment. Following an investigation by the manufacturer and Medicines and Healthcare products Regulatory Agency, a device-related cause could not be identified. In all three cases, manual CPR was immediately recommenced. In the fourth case, skin breakdown at the device compression point was noted.

There were a number of protocol deviations during the study, including the 28 participants randomised to mech-CPR who did not receive the intervention (Fig. 1). Three participants were retrospectively identified as having been ineligible at the point of randomisation: two of whom were in a shockable rhythm and one where an insufficient number of trained team members were present. All were included in the analysis in accordance with intention-to-treat principles. There were two cases in which envelopes were used out of sequence.

## Discussion

In this randomised feasibility trial comparing mech-CPR with manual-CPR in the hospital setting, we recruited 127 patients over a two-year period across five hospitals. We recruited 55% of potentially eligible patients. We observed effective deployment of mech-CPR devices and delivery of high-quality CPR across both study arms. Overall, 30-day survival was 4.7%. Due to lower than planned recruitment, we decided that it would not be informative to either statistically compare groups or make use of the Cocks and Torgerson approach.^24^

Our rationale for undertaking a feasibility trial reflects limited experience in both the UK and internationally of undertaking trials of intra-arrest interventions in the setting of in-hospital cardiac arrest, and the challenge of implementing a new health technology.^14^ Whilst many UK hospitals own mechanical chest compression devices, use is typically limited to specific locations, such as the emergency department or cardiac catheter laboratory.^12^

Our trial demonstrated the feasibility of 24/7 recruitment to a randomised controlled trial of an intra-arrest intervention across three hospital sites. In the remaining two sites, 24/7 recruitment was precluded by frequent changes in cardiac arrest team composition and the associated need to train a large number of individuals in device use and trial procedures. This is an important finding that will inform the design of future in-hospital trials.

In the specific context of a mech-CPR trial, we identified three key challenges that would likely preclude progression to an effectiveness trial, namely patient outcome; CPR quality; and overall recruitment. Firstly, for patient outcome, we observed a lower than expected hospital survival rate. Study recruits were patients in a non-shockable rhythm that had not responded to initial resuscitation measures. The implication of a low event rate is marked inflation of the sample size required to reliably detect a difference between study arms. For example, based on a baseline 30-day survival rate of 4.7%, a sample size of over 20,000 patients would be required to detect a small 1% difference in 30-day survival at 90% power and a significance level of 0.05.

Our original survival projection was based on registry data which reported a hospital survival rate of approximately 10% in patients that present in a non-shockable rhythm, compared with 45% in patients in a shockable rhythm.^1^, ^27^ Our observed 30-day survival rate of 4.7% is likely explained by a combination of our target population (patients in a non-shockable rhythm) and the timing of the intervention. Randomisation occurred several minutes after arrest onset, by which point patients had failed to respond to immediate treatments, such as chest compressions, ventilation and oxygenation, and drug therapy.

Recruitment of patients earlier in their cardiac arrest would have resulted in a higher survival rate.^28^, ^29^ In studies of intra-arrest interventions, time to intervention is an important determinant of outcome. For example, an analysis of the PARAMEDIC-2 trial found that adrenaline was most effective when given early in the cardiac arrest.^30^ Cardiac arrest observational studies are subject to resuscitation time bias as patients with longer cardiac arrests are most likely to receive intra-arrest interventions, such as drugs, advanced airways and mech-CPR.^31^ This bias highlights the importance of, wherever feasible, robustly testing treatments in a randomised controlled trial.

In this trial, recruitment of patients earlier in the arrest would have been challenging to achieve and reduced the reduced the trial’s external validity. Our approach was to strategically locate 2–3 devices across each study site to best reflect practice in hospitals that already use mechanical chest compression devices. Arrival of the device at the arrest was required for randomisation. Our median reported time to randomisation was six minutes, although, as this was based on clinician recollection of events, this likely reflects a best-case estimate.^32^ A deployment model in which a device is located in each clinical area would substantially increase cost, but may have limited effect on time-to-deployment. For example, in an observational study of mechanical device use in the cardiac catheter laboratory where the device was immediately available, median time to device use was 7.4 min.^33^

Secondly, the primary process by which it is proposed mechanical chest compression devices might improve outcome in cardiac arrest is through the optimisation of chest compression delivery. This reflects evidence that delivery of in-hospital CPR is often sub-optimal.^3^, ^34^ A key risk of mechanical chest compression device use are the pauses associated with deployment.^35^ Our data on pauses during device deployment compare favourably with published studies, including those in highly optimised systems.^18^, ^19^ However, in contrast to previous studies, the high quality of CPR that we observed in the manual chest compression arm meant that we did not observe any separation of trial arms in relation to CPR quality.^18^ Two of the five study hospitals routinely used real-time audiovisual CPR feedback which exceeds the rate reported in the literature.^36^

Thirdly, our overall recruitment was lower than anticipated. This was attributable to a number of system and patient factors, including the challenge of delivering 24/7 recruitment at two of our sites, the incidence of patients in shockable rhythm, and the decision to require two clinicians present trained in device use. These are important issues to consider when designing future trials.

Our study has a number of limitations. Firstly, despite the efforts of site teams, we were unable to achieve our planned sample size. This precluded key planned analyses, although we were able to base our decision that an effectiveness trial is not feasible on other findings. Secondly, we pragmatically selected study sites based on their willingness to participate. We do not know how representative these sites are of other UK hospitals. Across these sites, there was variability in use of real-time audiovisual feedback and previous experience with mech-CPR, which reflects variability across UK hospitals.^12^ Thirdly, compliance in the mech-CPR arm was 72%. Non-compliance was typically due to ROSC, but a number of other reasons were recorded including a decision that ongoing resuscitation was futile.

In this multi-centre feasibility randomised controlled trial, we identified specific challenges that preclude progression to an effectiveness trial of mech-CPR. These challenges were predominantly attributable to the challenge of implementing a new technology safely in the context of a clinical trial. Our findings demonstrate that recruitment to a randomised controlled trial of an intra-arrest intervention is feasible and highlight key issues that will require consideration in designing trials of other interventions.

## Funding

This research is funded by an NIHR Post-Doctoral Research Fellowship (PDF 2015-08-109). A funding condition was the use of an unequal randomisation ratio. Otherwise, the funder had no role in the design of the study, data collection, data analysis and interpretation, or the writing of the manuscript. GDP is supported as an NIHR Senior Investigator and by the NIHR Applied Research Centre (ARC) West Midlands, UK. The views expressed are those of the author(s) and not necessarily those of the NIHR or the Department of Health and Social Care.

Some equipment support was provided by the manufacturer (Jolfe AB/Stryker, Lund, Sweden) of the LUCAS mechanical chest compression device. The company had no role in the design of the study, data collection, data analysis and interpretation, or the writing of the manuscript.

## Conflicts of interest

GDP is an editor of Resuscitation. The remaining authors have no conflicts of interest to declare.

## CRediT authorship contribution statement

**Keith Couper:** Conceptualization, Methodology, Investigation, Writing - original draft, Writing - review & editing, Funding acquisition. **Tom Quinn:** Conceptualization, Methodology, Investigation, Writing - review & editing, Funding acquisition. **Katie Booth:** Investigation, Formal analysis, Writing - review & editing. **Ranjit Lall:** Conceptualization, Methodology, Investigation, Formal analysis, Writing - review & editing, Funding acquisition. **Anne Devrell:** Conceptualization, Methodology, Investigation, Writing - review & editing, Funding acquisition. **Barry Orriss:** Investigation, Writing - review & editing. **Scott Regan:** Project administration, Writing - review & editing. **Joyce Yeung:** Investigation, Writing - review & editing. **Gavin D. Perkins:** Conceptualization, Methodology, Investigation, Writing - review & editing, Funding acquisition.
