# Supplementary material for: Mechanical versus manual chest compressions in the treatment of in-hospital cardiac arrest patients in a non-shockable rhythm: A multi-centre feasibility randomised controlled trial (COMPRESS-RCT)
Source: Resuscitation. 2021 Jan;158:228–35. doi: 10.1016/j.resuscitation.2020.09.033 (PMC7790762; doi:10.1016/j.resuscitation.2020.09.033)
Supplement: Supplementary file 1 [file mmc1.docx]

COMPRESS-RCT Supplementary Information

Table S1: list of secondary outcomes

|  | Outcome | Time-point | Definition |
| --- | --- | --- | --- |
| Study feasibility outcomes | | | |
|  | Proportion of patients randomised outside of weekday daytime hours | Whole trial | Weekday daytime hours defined as Monday- Friday 08:00-19:59. |
|  | Device deployment time | During device deployment | Pause in chest compressions associated with device deployment |
|  | Proportion of patients/ consultees providing agreement to ongoing study participation | Whole trial | - |
|  | Proportion of patients with adequate blinding | Discharge, six-months | - |
|  | Proportion of patients with complete follow-up data | Discharge, six-months | - |
|  | Proportion of patients with analysable chest compression quality data. | Whole trial | - |
| Patient outcomes | | | |
|  | Return of spontaneous circulation | Cardiac arrest event | Return of a spontaneous circulation for at least twenty minutes. |
|  | Survival | Hospital discharge, 30-days, 6-months |  |
|  | Survival with good neurological outcomes measured with cerebral performance category (CPC) | Hospital discharge | Defined as a CPC of 1 or 2 or return to baseline (pre-admission) |
|  | Survival with good neurological outcomes measured with Modified Rankin Score (mRS) | Hospital discharge, 6-months | Defined as a mRS of 0-3 or return to baseline (pre-admission) mRS |
|  | Quality of life- measured using EQ-5D-5L | Hospital discharge, 6-months |  |
|  | Quality of life- measured using short form 12 | Hospital discharge, 6-months |  |
| Process outcomes | | | |
|  | Cardiopulmonary resuscitation (CPR) quality (chest compression rate, chest compression depth, flow-fraction, pre-shock pause, post-shock pause, peri-shock pause). |  |  |
| Safety outcomes | | | |
|  | Device related adverse events |  |  |

Table S2: Additional study feasibility outcomes

|  | Outcome |
| --- | --- |
| Proportion of patients with adequate blinding- discharge | 100% (97.5% CI 15.8 to 1)†  n=2 of 2 |
| Proportion of patients with adequate blinding- six-months | 100% (97.5% CI 15.8 to 1)†  n=2 of 2 |
| Proportion of patients with complete follow-up data- discharge | 40.0% (95% CI 5.3 to 85.3)  n=2 of 5 |
| Proportion of patients with complete follow-up data- six-months | 50.0% (95% CI 6.8 to 93.2)  n=2 of 4 |
| †- One-sided confidence interval | |

Table S3: Defibrillation data

|  | Mech-CPR  (n=99) | Man-CPR  (n=28) | All cases  (n=127) |
| --- | --- | --- | --- |
|  |  |  |  |
| Received defibrillation- n(%) | 17 (17.2%) | 2 (7.1%) | 19 (15.0%) |
| Number of shocks delivered to individuals that were defibrillated- median (IQR)† | 1 (1-2) | 1 (1-18) | 1 (1-2) |
| Pre-shock pause- median (IQR)‡ | 1.6 (0-4.9) | 1.7 (1.6-1.8) | 1.6 (0.5-2.6) |
| Post-shock pause- median (IQR)† | 1.7 (1.3-3.4) | 1.8 (1.5-2.1) | 1.7 (1.3-3.4) |
| Peri-shock pause- median (IQR)‡ | 3.3 (0-6.3) | 3.5 (3.1-3.9) | 3.3 (2-5.2) |
| †Data available for 15 participants (13 mechanical; 2 manual).  ‡Data available for 13 participants (11 mechanical; 2 manual). | | | |

Table S4: Length of stay and quality of life outcomes

|  | | Mech-CPR  (n=99) | Man-CPR  (n=28) | All cases  (n=127) |
| --- | --- | --- | --- | --- |
| Length of stay- days- mean (95% CI)† | |  |  |  |
|  | Critical care | 9.2 (3.8 - 14.7) | 3.6 (-0.6 - 7.8) | 7.2 (3.5 – 11.0) |
|  | Hospital | 33.0 (-19.2 – 85.2) | 16 (-) | 28.8 (-1.7 – 59.2) |
| Quality of Life- EQ-5D-5L health score- mean (SD)‡ | |  |  |  |
|  | Discharge | 53.0 (-99.5 – 205.5) | - | 53.0 (-99.5 – 205.5) |
|  | 6-months | 50 (-77.1 – 177.1) | - | 50 (-77.1 – 177.1) |
| Quality of Life- SF-12 – mean (SD)‡ | |  |  |  |
|  | Physical score – discharge | 35.4 (-3.7 – 74.4) | - | 35.4 (-3.7 – 74.4) |
|  | Mental score– discharge | 38.1 (-45.1 – 121.3) | - | 38.1 (-45.1 – 121.3) |
|  | Physical score- 6-months | 27.4 (5.7 – 49.1) | - | 27.4 (5.7 – 49.1) |
|  | Mental score- 6-months) | 33.7 (-177.9 – 245.2) | - | 33.7 (-177.9 – 245.2) |
| †- Data from 14 participants for critical care length of stay (9 mechanical; 5 manual) and 4 participants for hospital length of stay (3 mechanical; 1 manual)  ‡- Data available for two participants | | | | |
